# Modulation of the regenerative microenvironment within acellular nerve allografts using tacrolimus improves nerve regeneration

**DOI:** 10.4103/NRR.NRR-D-25-01014

**Published:** 2026-02-28

**Authors:** Jesús A. Acevedo Cintrón, Jonathon Blake Schofield, Daniel A. Hunter, Pranay Singh, Alexa M. Negrón Morales, Lauren Schellhardt, Susan E. Mackinnon, Matthew D. Wood

**Affiliations:** Division of Plastic Surgery, Department of Surgery, Washington University School of Medicine, St. Louis, MO, USA

**Keywords:** acellular nerve allograft, immunosuppression, macrophages, microenvironment, nerve gap, osteopontin (SPP1), peripheral nerve regeneration, Schwann cells, tacrolimus (FK506), transcriptome analysis

## Abstract

Acellular nerve allografts have been used as an alternative to reconstruct nerve gaps. However, regeneration and recovery using long acellular nerve allografts (> 3 cm) is poor in comparison to short acellular nerve allografts (< 3 cm). To understand why long acellular nerve allografts have limited regeneration, we focused on identifying differences in the microenvironment of short (2 cm) and long (4 cm) acellular nerve allografts by comparing the transcriptional profile of these acellular nerve allografts. After repairing the sciatic nerve of Lewis rats using either short or long acellular nerve allografts, we found that the proximal and mid-distal graft regions of long acellular nerve allografts are characterized by an upregulation of metabolic and immune pathways and downregulation of regenerative processes in comparison to the short acellular nerve allografts. Based on these results, we modulated the regenerative and immune microenvironment of long acellular nerve allografts using tacrolimus (FK506). Histomorphometric and muscle force analysis revealed that FK506 increases the number of axons and improves recovery of motor function across long acellular nerve allografts. Transcriptome analysis of the mid-distal graft region of long acellular nerve allografts from animals treated with FK506 revealed upregulation of regenerative pathways and downregulation of immune processes, specifically related to T cell activity. Additionally, FK506 altered the number of macrophages and Schwann cells in the long acellular nerve allografts. From the transcriptome analysis, we identified FK506 upregulates expression of *Spp1* (osteopontin) which promotes regeneration of motor neurons after injury. Experiments on cultured Schwann cells revealed that FK506 increases mRNA expression of *Spp1*. Our data show the development of a degenerative and immune microenvironment within long acellular nerve allografts and demonstrate that FK506 can modulate this microenvironment to improve nerve regeneration across these long acellular nerve allografts.

## Graphical abstract

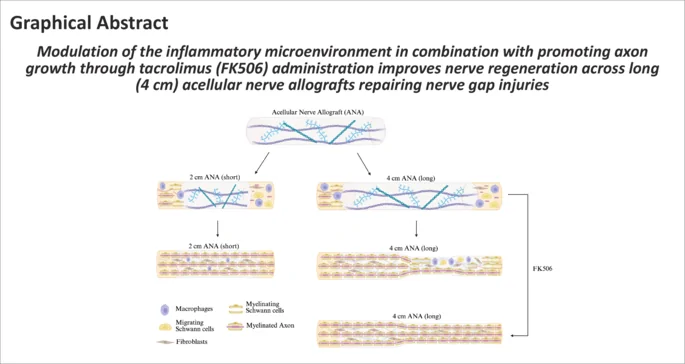

## Introduction

Nerve injuries continue to pose a challenge in the clinic with approximately 30,000 new cases annually in the US (Taylor et al., 2008; Padavano et al., 2020; Bellaire et al., 2021). Of all nerve injuries, neurotmesis is considered the most severe type of nerve injury resulting in significant morbidity and long-term disability. These injuries require surgical intervention and oftentimes the use of a graft to bridge the gap between nerve ends to facilitate regeneration (Pripotnev et al., 2022). Autologous nerve grafts are considered the gold standard of grafts due to their ability to promote regeneration across gaps (Ray and Mackinnon, 2010; Moore et al., 2011; Boriani et al., 2019; Pan et al., 2020a; Lans et al., 2023; Johnson et al., 2024; Saffari, 2024). However, the use of autografts can be limited by the size and length of the donor nerve and the added surgical time to harvest (Ray and Mackinnon, 2010; Moore et al., 2011; Gao et al., 2015; Pan et al., 2020a; Johnson et al., 2024). Due to these limitations, alternatives to repair nerve gaps, such as nerve guidance conduits or decellularized nerve grafts, have been proposed. Acellular nerve allografts (ANAs) are decellularized nerves that are obtained from cadavers and processed with detergents to remove immunogenic components but maintain the extracellular matrix (ECM) of the nerve (Pan et al., 2020a; Bellaire et al., 2021; Peters et al., 2023). The presence of the ECM serves as scaffold for host cells to repopulate the ANA and promote regeneration. The advantages of using ANAs are their immediate availability and shared surgical handling of nerve autografts. However, the main disadvantage of ANAs is that while regeneration across ANAs less than 3 cm in length is generally robust, regeneration across ANAs greater than 3 cm in length can be limited (Ray and Mackinnon, 2010; Moore et al., 2011; Leckenby et al., 2020; Peters et al., 2023; Johnson et al., 2024; Saffari, 2024).

**Highlights**

• Long *versus* short acellular nerve allografts have inferior nerve regeneration when used to reconstruct nerve gap injuries.

• Long allografts are characterized by an upregulation of metabolic and immune pathways and downregulation of regenerative processes compared with short allografts.

• Administration of tacrolimus (FK506) can improve nerve regeneration across long allografts.

• Administration of tacrolimus correlated with decreased pro-inflammatory immune pathways and upregulated regenerative process pathways.

Multiple rodent studies have compared the microenvironment of ANAs to understand why ANAs longer than 3 cm (i.e., long ANAs) fail to promote nerve regeneration (Saheb-Al-Zamani et al., 2013; Poppler et al., 2016; Pan et al., 2019; Acevedo Cintron et al., 2024). These studies showed a significant reduction in the number of axons in the mid-distal graft region of long ANAs in comparison to ANAs shorter than 3 cm (i.e., short ANAs). The significant reduction in the number of axons in the mid-distal graft region of long ANAs, in comparison to short ANAs, coincided with an increase in the number of senescent Schwann cells (SCs) (Saheb-Al-Zamani et al., 2013; Poppler et al., 2016). Subsequent studies suggested the axonal arrest across long ANAs is caused by the lack of permissive environment for regeneration to occur and not the inability of axons to grow across long distances (Poppler et al., 2016; Pan et al., 2019). Therefore, understanding why and how the regenerative microenvironment within a nerve gap forms to facilitate successful axon regeneration could be pivotal to improving regeneration across long nerve gaps.

Studies considering the early stages of nerve regeneration that lead to the formation of the regenerative microenvironment between a short nerve gap have been consequently. After an unrepaired neurotmesis, Cattin et al. (2015) showed that the initial stages of regeneration are characterized by the formation of an endogenous scaffold that is infiltrated by macrophages. Due to the hypoxic environment of this scaffold, macrophages secrete pro-angiogenic vascular endothelial growth factor to promote angiogenesis within the scaffold. These newly formed blood vessels are then used by SCs to migrate into and across the scaffold allowing axons to subsequently grow across the gap. Disruption of these processes leads to poor and delayed regeneration.

In the context of ANAs, a similar process occurs, where macrophages are amongst the first cells to infiltrate and repopulate the ANAs followed by the formation of blood vessels. These processes are then followed by SC migration into the ANA and axonal growth across the graft (Pan et al., 2019, 2020a, b). In our previous study, disruption of macrophage infiltration or angiogenesis in short ANAs leads to poor and delayed regeneration (Pan et al., 2020b). These studies highlight the key roles of non-neuronal cells such as macrophages and endothelial cells play in forming a permissive microenvironment for axons to grow across acellular scaffolds.

With previous studies demonstrating the critical role of macrophages in regeneration after injuries, follow up studies have focused on the role of the broader immune system and inflammation in orchestrating regeneration across ANAs. These studies have shown differences in the inflammatory and cell microenvironment as time progresses (Pan et al., 2019; Acevedo Cintron et al., 2024). Early in the regenerative process (before 4 weeks post-surgery), the mid-graft of short ANAs is characterized by an increase in the number of macrophages and T cells, and increased expression of cytokines in comparison to long ANAs (Pan et al., 2020c). However, after 4 weeks, the mid-distal graft region of long ANAs is characterized by a significant increase in the expression of pro-inflammatory cytokines, changes in blood vessel morphology, but no difference in the number of macrophages compared with short ANAs (Acevedo Cintron et al., 2024). Taken together, these studies suggest differences in the inflammatory and cellular microenvironment within ANAs as regeneration progresses that could be central to differences in regeneration based upon ANA length. Specifically, short ANAs are characterized by an early inflammatory “wave” that resolves allowing for regeneration. However, in the long ANAs, the early inflammatory wave is delayed and unresolved, possibly hampering regeneration across long ANAs.

Since the microenvironment in long ANAs becomes pro-inflammatory, we are interested in observing if using an immunosuppressant to reduce the inflammatory response improves nerve regeneration across long ANAs. Previous studies have shown lack of macrophages and T cells disrupts and delays nerve regeneration across short ANAs (Pan et al., 2019, 2020b). However, it is unknown if immunosuppression negatively impacts regeneration across long ANAs. Tacrolimus (FK506) is an immunosuppressant that has been widely used in preventing solid organ transplant rejection (Allison, 2000; Barbarino et al., 2013). FK506 immunosuppresses by binding to its receptor FK506-binding protein 12 (FKBP12) preventing calcineurin activation (**Additional Figure 1A**). Calcineurin inactivation prevents dephosphorylation of nuclear factor of activated T cells (NFAT), thus preventing NFAT translocation to the nucleus. This further prevents T cell activation, proliferation, and cytokine product, such as interleukin-2 (Mouzaki et al., 1992; Rao et al., 1997; Khanna et al., 1999; Allison, 2000; Tsuda et al., 2012; Barbarino et al., 2013). It is also hypothesized FK506 promotes polarization to an anti-inflammatory phenotype in macrophages (Bai et al., 2010; Kannegieter et al., 2017). However, the exact mechanism by which FK506 induces this is unknown.

In addition to its immunosuppressive properties, FK506 improves nerve regeneration after nerve injury by promoting axonal extension, SC proliferation, secretion of neurotrophic factors, and reduction of scarring within nerve tissue (Gold et al., 2004; Konofaos and Terzis, 2013; Que et al., 2013; Davis et al., 2019). One of the proposed mechanisms by which FK506 enhances regeneration is by binding to FKBP12 on SCs and axons enhancing phosphorylation of growth-associated protein-43 and activation of transforming growth factor β1 (Lyons et al., 1995; Saffari et al., 2019). It is hypothesized that activation of these pathways leads to increased expression of neurotrophic factors and growth cone motility (Saffari et al., 2019). Another possible mechanism by which FK506 promotes regeneration, is by binding to a different receptor, FKBP52, on axons, causing disassembly of the steroid receptor complex and activation of the ERK signaling pathway leading to neurite elongation (**Additional Figure 1B**; Gold, 1999; Gold et al., 1999; Gold and Zhong, 2004; Quinta and Galigniana, 2012; Saffari et al., 2019). However, it is unknown if these are the only and most prominent mechanisms by which FK506 promotes regeneration.

Although most studies have focused on the efficacy of FK506 in promoting regeneration using crush and direct repair injury models, there is limited information if and how FK506 affects regeneration across decellularized grafts *in vivo*. Recently, however, a study showed low doses of FK506 promote regeneration similar to an autograft across 1-cm cold and cryo-preserved allografts (Kim et al., 2024). Similarly, *in vitro* studies using chemically decellularized grafts attached to dorsal root ganglion (DRG) demonstrated FK506 increases neurite extension across these grafts (Saffari et al., 2021).

Due to the cellular complexity of regenerating ANAs, our current study aimed to identify differences between the microenvironment of short and long ANAs using transcriptome analyses before the development of a pro-inflammatory microenvironment in long ANAs. Our study also considered if FK506 modulated the inflammatory and regenerative microenvironment within long ANAs through transcriptome analyses, and whether this would lead to subsequently improved nerve regeneration across these grafts.

## Methods

### Experimental design and animals

Commercially-available adult rats (200–250 g, Charles River Laboratories, Wilmington, MA, USA) were utilized for all experiments. Sprague–Dawley rats were used as nerve donors to generate ANAs. Lewis rats were used for the experimental group receiving ANAs. Surgical procedures and peri-operative care measures were conducted in compliance with the Association for Assessment and Accreditation of Laboratory Animal Care accredited Washington University Institutional Animal Care and Use Committee (IACUC; Animal Welfare Assurance #D16-00245; Protocol No. 22-0423) and the National Institutes of Health guidelines. All animals were housed in a central animal care facility and provided with food (PicoLab rodent diet 20, Purina Mills Nutrition International, St. Louis, MO, USA) and water *ad libitum.*

The studies included multiple independent sets of animals to measure a variety of outcome metrics. For groups, rat sciatic nerve was transected and repaired using either short (2 cm) or long (4 cm) ANAs. Studies using short and long ANAs assessed differences in the transcriptome profile between ANAs of different lengths 4 weeks post-surgery. Studies using FK506-treated and untreated animals with long ANAs assessed if and how FK506 affected regeneration across long ANAs. For this comparison, changes in the transcriptome profile of untreated long ANAs and FK506-treated long ANAs were assessed 4 weeks post-surgery using bulk-RNA sequencing. Additionally, in a separate set of animals, the extent of nerve regeneration and the quantity and morphology of blood vessels was assessed 8 weeks post-surgery using histology and histomorphometry. Another separate set of animals was used to assess the extent of nerve regeneration and functional recovery using muscle force measurements and mechanical sensitivity analysis 12 weeks post-surgery. Similar to our previous work (Acevedo Cintron et al., 2024), at the corresponding endpoints, where applicable, the harvested nerve tissue was divided into three regions: proximal graft (proximal 1/3 of ANA), mid-distal graft (distal 2/3 of ANA), and distal stump. The number of animals used per cohort are outlined in **Additional Table 1**.

**Additional Table 1:** Number of animals assigned per experiment

Cohort	Graft length	Animal number	Endpoint	Analysis
Lewis (M)	2 cm ANA	8	4 weeks	Bulk RNA-sequencing and immunohistochemistry
	4 cm ANA	8	4 weeks	Bulk RNA-sequencing and immunohistochemistry
Lewis (M)	4 cm ANA	7	4 weeks	Bulk RNA-sequencing and immunohistochemistry
	4 cm ANA + “Day 0” FK506	8	4 weeks	Bulk RNA-sequencing and immunohistochemistry
Lewis (M)	4 cm ANA	5	8 weeks	Histology/Histomorphometry
	4 cm ANA + “Pre-load” FK506	6	8 weeks	Histology/Histomorphometry
	4 cm ANA + “Day 0” FK506	6	8 weeks	Histology/Histomorphometry
Lewis (M)	4 cm ANA	8	12 weeks	Histology/Histomorphometry, muscle force and mechanical sensitivity
	4 cm ANA + “Pre-load” FK506	9	12 weeks	Histology/Histomorphometry, muscle force and mechanical sensitivity
	4 cm ANA + “Day 0” FK506	8	12 weeks	Histology/Histomorphometry, muscle force and mechanical sensitivity
	4 cm ANA + Cyclosporine	8	12 weeks	Histology/Histomorphometry
	4 cm ANA + geldanamycin	9	12 weeks	Histology/Histomorphometry

ANA: Acellular nerve allografts; FK506: tacrolimus.

### Surgical procedures

To generate ANAs, Sprague–Dawley rats were euthanized with an intraperitoneal injection of pentobarbital (150 mg/kg; Fresenius Kabi, Lake Zurich, IL, USA). Subsequently, the sciatic nerve was exposed proximally from the level of the exiting L5–L6 nerve roots at the cruciate ligament to the level of the sciatic nerve trifurcation distally. The length of the harvested grafts yielded 5–6 cm of nerve. The harvested nerves were decellularized (see below) and then trimmed to the appropriate length (2 or 4 cm) as necessary immediately before grafting procedures.

For the grafting of the processed nerves (**Additional Figure 2**), Lewis rats were anesthetized by subcutaneous injections of a ketamine (75 mg/kg; Forth Dodge Animal Health, Fort Dodge, IA) and dexmedetomidine (0.5 mg/kg; Pfizer Animal Health, Exton, PA, USA) cocktail. The right sciatic nerve was exposed by incising the skin and biceps femoris muscle **~**1–2 cm below the femur. The exposed sciatic nerve was then transected 4 mm proximal to the distal trifurcation. The grafts were sutured to repair the gap with 9-0 nylon. The grafted nerves were shaped into multiple loops (“S” shape), and the connective tissue surrounding the grafts was sutured into the caudofemoralis muscle with 11-0 nylon to anchor the graft to the wound area and prevent graft clumping. Following the repair, a two-layer muscle and skin closure was performed using a 6-0 vicryl and 4-0 nylon suture, respectively. Atipamezole hydrochloride solution (0.1 mg/kg; Modern Veterinary Therapeutics, Sunrise, FL, USA) was administered for anesthesia reversal and postoperative pain was managed using Buprenophrine SR (0.05 mg/kg; ZooPharm, Windsor, CO, USA). The animals recovered on a warming pad and were monitored for postoperative complications before being returned to the central animal care facility. Animals were monitored daily for signs of infection, distress, suture dehiscence, and/or lethargy. At the respective endpoints to harvest tissues, animals were euthanized (via pentobarbital as above) and procedures performed as just described above.

### Generation of acellular nerve allografts

Harvested nerves from rats were decellularized using a method described previously (Hudson et al., 2004; Poppler et al., 2016). Briefly, the nerves were repeatedly washed in deionized water followed by a sodium phosphate buffer containing sulfobetaine-16 (SB-16) and a combination of Triton X-100 and sulfobetaine-10 (SB-10). After processing, the ANAs were washed and stored in a 10 mM phosphate-buffered 50 mM sodium solution at 4°C until use (within 3 days).

### FK506 preparation and dosing

Animals transplanted with long (4 cm) ANAs were randomized to receive one of two dosing regimens of FK506 (“pre-load” or “Day 0” groups) or saline injections. The animals in the “pre-load” groups received daily subcutaneous administrations of FK506 (2 mg/kg subcutaneously; LC Laboratories, Woburn, MA, USA) starting 3 days preoperatively and continuing daily for the duration of the experiment (Udina et al., 2002; Sobol et al., 2003; Yang et al., 2003; Yan et al., 2012). Previous studies have shown that preoperatively dosing of FK506 results in substantial improvement of axonal regeneration (Lee et al., 2000; Snyder et al., 2006). Animals in the “day 0” groups received daily subcutaneous administration of FK506 for 5 consecutive days (except the first week when animals received injections every day) followed by 2 days with no FK506 administration, starting at the day of the surgery. This strategy was chosen to balance the potential benefits of FK506 while mitigating negative side effects. Animals in the untreated group, received daily saline injections starting 3 days preoperatively. Fresh aliquots of FK506 were made weekly using ethanol and polyethoxylated castor oil (Sigma-Aldrich, St Louis, MO, USA) as diluents and stored at –20°C until use. On the day of administration, the aliquots were diluted in 0.9% saline (Hospira Inc., Lake Forest, IL, USA). Due to the side effects of FK506, animals were monitored regularly for changes in weight and overall physical appearance (**Additional Figure 3**).

### Cyclosporine and geldanamycin preparation and dosing

Animals transplanted with long (4 cm) ANAs were randomized to receive daily subcutaneous injections of cyclosporine (10 mg/kg; LC Laboratories), geldanamycin (0.2 mg/kg; Thermofisher Scientific, Waltham, MA, USA) or saline starting on the day of surgery. In previous studies, this concentration of cyclosporine has been demonstrated to induce immunosuppression in rats (Bain et al., 1988; Midha et al., 1992). For geldanamycin, this concentration has been shown to promote regeneration in a pre-conditioned nerve injury model (Sun et al., 2012). After the first week, animals received daily injections for five consecutive days followed by 2 days with no drug administration for the duration of the experiments (12 weeks). Fresh aliquots of cyclosporine were prepared weekly by using ethanol and polyethoxylated castor oil (Sigma-Aldrich) as diluents and stored at –20°C until use. Similarly, fresh aliquots of geldanamycin were prepared weekly using Dimethyl sulfoxide (DMSO; Thermofisher Scientific) as diluent and stored at –20°C until use. On the day of administration, the aliquots were diluted in 0.9% saline (Hospira Inc.).

### RNA sequencing and analysis

Total RNA was extracted from harvested ANAs with QIAGEN RNAeasy Micro Kit (QIAGEN, Germantown, MD, USA) according to the manufacturer’s protocol. Total RNA from samples was sequenced on an Illumina NovaSeq X Plus (Novogene, Sacramento, CA, USA). Basecalls and demultiplexing were performed with Illumina’s DRAGEN and BCLconvert version 4.2.4 software (Novogene, Sacramento, CA, USA). RNA-seq reads were then aligned to the Ensembl release 101 primary assembly with STAR version 2.7.9a (Dobin et al., 2013). Gene counts were derived from the number of uniquely aligned unambiguous reads by Subread:featureCount version 2.0.3 (Liao et al., 2014). Isoform expression of known Ensembl transcripts was quantified with Salmon version 1.5.2 (Patro et al., 2017). Sequencing performance was assessed for the total number of aligned reads, total number of uniquely aligned reads, and features detected. The ribosomal fraction, known junction saturation, and read distribution over known gene models were quantified with RSeQC version 4.0 (Wang et al., 2012).

All gene counts were then imported into the R/Bioconductor package EdgeR and TMM normalization size factors were calculated to adjust for samples for differences in library size (Robinson et al., 2010). Ribosomal genes and genes not expressed in the smallest group size minus one samples greater than one count-per-million were excluded from further analysis. The TMM size factors and the matrix of counts were then imported into the R/Bioconductor package Limma (Ritchie et al., 2015). Weighted likelihoods based on the observed mean-variance relationship of every gene and sample were then calculated for all samples and the count matrix was transformed to moderated log 2 counts-per-million with Limma’s voomWithQualityWeights (Liu et al., 2015). The performance of all genes was assessed with plots of the residual standard deviation of every gene to their average log-count with a robustly fitted trend line of the residuals. Differential expression analysis was then performed to analyze for differences between conditions and the results were filtered for only those genes with Benjamini-Hochberg false-discovery rate (FDR) adjusted *P*-values less than or equal to 0.05. Volcano plots and MA plots representing differentially expressed genes (DEG) were generated using GraphPad Prism V10.3.1 (GraphPad Software, LLC, La Jolla, CA, USA).

For each contrast extracted with Limma, global perturbations in known Gene Ontology (GO) terms, MSigDb, and KEGG pathways were detected using the R/Bioconductor package GAGE to test for changes in expression of the reported log2 fold-changes reported by Limma in each term *versus* the background log2 fold-changes of all genes found outside the respective term (Luo et al., 2009). The R/Bioconductor package heatmap was used to display comparisons across groups of samples for each GO or MSigDb term with a Benjamini-Hochberg false-discovery rate (FDR) adjusted *P*-value less than or equal to 0.05 (Zhao et al., 2014). Perturbed KEGG pathways where the observed log 2 fold-changes of genes within the term were significantly perturbed in any direction compared with other genes within a given term with *P*-values less than or equal to 0.05 were rendered as annotated KEGG graphs with the R/Bioconductor package Pathview (Luo and Brouwer, 2013). Graphs representing the top five upregulated and downregulated GO terms, MSigDb terms, and KEGG pathways were generated using R studio V4.2.2.

### Immunohistochemistry of nerve

To assess FK506 mediated changes in the cellular composition of ANAs, macrophages and SC within the long untreated and FK506-treated ANAs at the 4-week endpoint were stained, imaged, and quantified using immunohistochemistry. After harvest, portions of the proximal and mid-distal grafts were immediately placed in 4% paraformaldehyde in phosphate-buffered saline overnight followed by immersion in 30% sucrose in phosphate-buffered saline (PBS) for 24–48 hours. The tissues were then placed in plastic cassettes oriented for cross-sections and frozen in OCT Compound (VWR, Radnor, PA, USA), then sectioned with a cryostat at 15 µm onto pretreated charged glass slides. Sections were rehydrated in PBS and blocked for 1 hour using a solution of 8% normal goat serum, 2% bovine serum albumin and 0.1% Triton X-100 diluted in PBS. After blocking, a solution containing the primary antibodies diluted in the blocking buffer was applied to the sections and incubated at 4°C overnight. Primary antibodies were used to stain macrophages (CD68) and SCs (S100) followed by PBS wash and stain for the appropriate fluorochrome-conjugated secondary antibodies for 1 hour at room temperature. Concentrations are outlined in **Additional Table 2**. Slides were then washed with PBS and sections were mounted with Fluoroshield mounting medium with 4′,6-diamidino-2-phenylindole (DAPI; Abcam, Boston, MA, USA) and imaged using a Fluoview F100 confocal microscope and acquisition system (Olympus, Waltham, MA, USA) at 200× (20× water immersion objective) overall magnification. For macrophage and SC quantification, percent area was measured using ImageJ (version 1.51, National Institutes of Health, Bethesda, MD, USA), where positive marker pixels are totaled within a standardized field. Six sections per graft region and group were analyzed and averaged using ImageJ to obtain values per animal (*n* = 1).

**Additional Table 2:** Antibodies used for immunohistochemistry

	Antibodies	Host	Supplier	Cat#	Dilution
Primary antibodies	S100	Rabbit	DAKO, Carpinteria, CA, USA	GA50461-2	1:5
	CD68	Mouse	Bio-Rad, Hercules, CA, USA	MCA341R	1:500
	Alexa Fluor 633	Phalloidin	Thermofisher Scientific, Waltham, MA, USA	A22284	1:500
Secondary antibodies	Anti-mouse Alexa Fluor 488	Goat	Thermofisher Scientific	A32723	1:1000
	Anti-rabbit Alexa Fluor 555	Goat	Thermofisher Scientific	A21428	1:1000

### Histology and histomorphometry of nerve

To evaluate nerve regeneration, histology incorporating histomorphometric analyses of the nerve was performed as previously described (Hunter et al., 2007, 2020). Collected nerves were fixed via immersion in 3% glutaraldehyde in phosphate buffer and then trimmed into pieces corresponding to the spatial region of nerve or graft. The samples were then fixed in 1% osmium tetroxide (Sigma-Aldrich) and serially dehydrated using various concentration of ethanol. The tissues were then embedded in Araldite 506 epoxy resin and cross-sectioned using an ultra-microtome at 1.5 µm sections. The sections were then counterstained with 1% toluidine blue and imaged for analyses at 1000× overall magnification on a Leitz Laborlux S microscope (Leica Microsystems, Deerfield, IL, USA). For analysis, a semi-automated digital image-analysis system linked to morphometry macros developed for peripheral nerve analysis (Clemex Vision Professional, Clemex Technologies, Longueuil, Quebec) was used. Six to eight random fields from two or three different histological sections (accounting for different areas across the length of the nerve) were imaged resulting in 75%–80% of the cross-sectional area analyzed. Binary histomorphometry analysis of the digitized information based on gray and white scales allowed measurements of total fascicular area and total number of myelinated axons in the relevant nerve sections. Manual quantification of myelinated axons across multiple randomly selected fields per nerve permitted calculation of the percent of myelinated axons, area covered by myelinated axons (µm^2^), density of myelinated axons (per mm^2^), percent of myelinated axons debris, average size area of myelinated axon, average size area of axon, and average size area of myelin. Manual quantification of blood vessels permitted calculation of the density of blood vessels in given fields (per mm^2^). To assess blood vessel morphology, a ratio representing the area of the blood vessel lumen divided by the total area of the blood vessel was used to compare morphological changes between blood vessels. The numbers for both analysis of nerve regeneration and microvasculature were averaged across the images per animal (*n* = 1).

### Muscle force analysis

Twelve weeks postoperatively, sciatic nerve function was assessed by examining the evoked motor response in reinnervated gastrocnemius muscle. All animals were re-anesthetized and the sciatic nerve proximal to the ANA was isolated using a gluteal-splitting approach. The common tendinous attachment of the gastrocnemius and soleus was surgically isolated, and the soleus muscle was transected. The distal gastrocnemius was attached to a 50-N thin-film load cell (S100; Strain Measurement Devices, Meriden, CT, USA) using a steel S hook. A custom-designed force-measurement jig (Red Rock Laboratories, St. Louis, MO, USA) was used to immobilize the knee and deliver cathodic, monophasic electrical impulses with constant current (duration 200 microseconds, frequency 0 to 200 Hz, current amplitude 0 to 2 mA) using a single-channel isolated pulse simulator (Model 2100; A-M Systems, Carlsborg, WA, USA) to the sciatic nerve proximal to the ANA using bipolar silver wire electrodes.

Twitch contractions measured using a custom force recording system were utilized to determine the optimal stimulus current amplitude and optimal muscle length for isometric force production in the gastrocnemius muscle. All subsequent isometric force measurements were made at these parameters. Single-twitch contractions were recorded, and peak twitch force was calculated. Tetanic contractions were recorded at increasing frequencies (5 to 200 Hz) of stimulation. Maximum isometric tetanic force was subsequently calculated from the resulting force traces.

### Relative muscle mass analysis

After the completion of the muscle force analysis, animals were humanely killed. The gastrocnemius muscle was harvested from the experimental and contralateral sides. Wet muscle weight was recorded on each side, and the ratio of the ipsilateral to contralateral muscle weight was calculated.

### Mechanical sensitivity analysis

Mechanical sensitivity analysis was performed pre-operatively, 4, 8, and 12 weeks post-surgery to observe any changes in mechanical sensitivity as regeneration progresses. Animals were placed under ventilated Plexiglas boxes on an elevated meshed metal grid for 1 hour before measuring paw-withdrawal thresholds with the use of von Frey filaments (2.56–60 g) (TouchTest; North Coast Medical Inc, Morgan Hill, CA, USA). Analysis of Von Frey data was performed based on Chaplan et al., where values represent 50% of the withdrawal threshold or the threshold that the animal will respond 50% of the time (Dixon, 1980; Chaplan et al., 1994).

### Gene expression analysis of osteopontin (*Spp1*) on cultured Schwann cells

We performed analysis of gene expression of *Spp1* on SCs based on the bulk RNA sequencing results showing *Spp1* was upregulated in conditions where regeneration was improved. Primary SCs derived from rats were placed on poly-L-lysine coated 12 wells plates at a concentration of 10,000 cells/well with SC Medium (ScienCell Research Laboratories, Carlsbad, CA, USA). After 24 hours, SCs were treated with SC media (untreated group), 0.02% of DMSO (vehicle CTL group), 1 μg/mL, or 10 μg/mL of FK506 for 48 hours. Each group was represented with six samples. For SC treatment, FK506 was initially diluted with DMSO followed by dilution with SC media. After 48 hours, media containing the drug was replaced with fresh 1× PBS, the cells were collected and frozen and immediately placed in Trizol (Life Technologies, Carlsbad, CA, USA) in liquid nitrogen. The cells were then thawed and RNA extracted using Direct-zol^TM^ RNA MicroPrep w/TriReagent (Zymo Research, Irvine, CA, USA) according to the manufacturer’s instructions. Extracted RNA concentration was measured using a NanoDrop 1000 Spectrophotometer (Thermofisher Scientific) and adjusted for cDNA preparation. The cDNA was generated with SuperScript II reverse transcriptase (Thermofisher Scientific). To quantify gene expression, quantitative reverse transcription-polymerase chain reaction (qRT-PCR) was performed using a Step One Plus thermocycler (Thermofisher Scientific) and Taqman Master Mix (Thermofisher Scientific) reagents with a specific oligonucleotide prime for *Spp1* (**Additional Table 3**). The qRT-PCR conditions were a hot start at 95°C for 10 minutes, followed by 50°C for 2 minutes, 95°C for 15 seconds and 60°C for 1 minute intervals, repeated for 40 cycles. The gene expression of the selected genes was normalized to an internal control gene (*Actb*). Data was analyzed using Step One Software v3.0.1 (Thermofisher Scientific). Analysis of each well was considered a replicate for each group (*n* = 1).

**Additional Table 3:** Quantitative reverse transcription-polymerase chain reaction primers used in this study

Gene name	Thermofisher Assay ID	RefSeq
*Spp1*	Rn00681031_m1	NM_012881.2
*Actb*	Rn00667869_m1	NM_031144.3

### Immunohistochemistry of osteopontin (*Spp1*) on cultured Schwann cells

Primary rat SCs were placed on poly-L-lysine coated coverslips inside 24-well plates at a concentration of 5000 cells/well with SC Medium (ScienCell Research Laboratories). After 24 hours, SCs were treated with SC media, DMSO, or FK506 similar to the process used to measure the gene expression analysis of *Spp1* (see above). After the PBS wash, 4% paraformaldehyde in PBS was added to the wells for 20 minutes and blocked for 1 hour with the same buffer used for nerve tissue (see above). After blocking, anti-Osteopontin antibody was diluted in the blocking buffer, applied to the coverslips and incubated at 4°C overnight. Incubation of primary antibody was followed by PBS wash and stain for the appropriate fluorochrome-conjugated secondary antibodies for 1 hour at room temperature. Slides were then washed with PBS and sections were incubated with Alexa Fluor 633 Phalloidin for 20 minutes (Thermofisher Scientific). Antibodies concentrations are outlined in **Additional Table 2**. Coverslips were then mounted onto pretreated charged glass slides using Fluoroshield mounting medium with DAPI (Abcam) and imaged using a Fluoview F100 confocal microscope and acquisition system (Olympus) at 600× (60× oil immersion objective) overall magnification. For Osteopontin quantification, percent area per cell was measured using the ImageJ macro, where positive marker pixels are totaled within a standardized field. Three areas per coverslip were analyzed and averaged using ImageJ to obtain values per coverslip (*n* = 1).

### Statistical analysis

Statistical analyses were performed using GraphPad Prism V10.3.1. Each animal and its nerve or cultured SC well represented an ‘*n*’ value. Data were represented as mean ± standard deviation with individual values. Data sets were tested for normality using the Shapiro-Wilk test. Outliers within each group were identified using the ROUT method with *Q* = 1% and removed from analysis. Student’s *t*-test was performed for comparison between two groups with Gaussian distribution. Welch’s correction was included if the standard deviation between two groups was significantly different. For comparison between two groups with a non-Gaussian distribution, the Mann-Whitney *U* test was performed. For comparison between three groups with Gaussian distribution, one-way analysis of variance with the Tukey’s comparisons for *post hoc* analysis between groups was used. Welch’s correction was included if the standard deviation between groups was different. For comparison between three groups with a non-Gaussian distribution, Kruskal-Wallis test with the Dunn’s comparison for *post hoc* analysis between groups was used. Spearman correlation analysis was performed to observe monotonic relationships between two variables represented with the correlation coefficient (R). A significance level of *P* < 0.05 was used for all comparisons.

## Results

### The transcriptome differs in the proximal graft region of acellular nerve allografts based on acellular nerve allograft length

**Figure 1:**
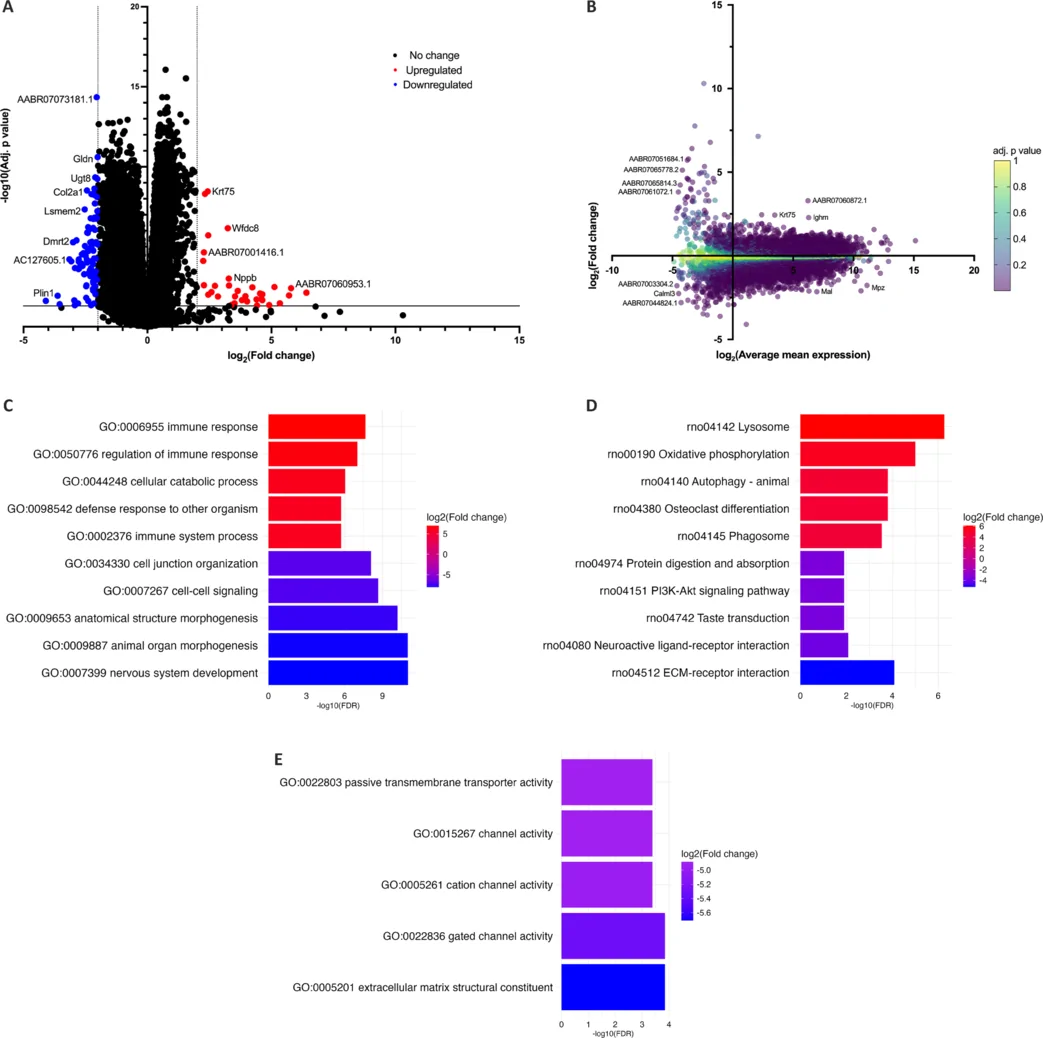
The proximal graft of long *versus* short ANAs is characterized by upregulation of immunological and catabolic processes, and downregulation of pathways related to nerve regeneration. (A) Volcano plot representing genes expressed in the long *versus* short ANAs. Black dots represent non-DEGs, blue dots represent down-regulated DEGs and red dots upregulated DEGs in long *versus* short ANAs. (B) Log ratio *versus* average (MA) plot representing long *versus* short ANAs. Classification of top five upregulated and downregulated (C) GO biological processes, (D) KEGG pathways, and (E) molecular function for long *versus* short ANAs. *n* = 8 per group, where *n* is each data point representing an animal. ANAs: Acellular nerve allografts; DEGs: differentially expressed genes; GO: gene ontology; KEGG: Kyoto Encyclopedia of Genes and Genomes.

Based on previous work showing inferior nerve regeneration across long *vs.* short ANAs (Acevedo Cintron et al., 2024), transcriptional analysis of short and long ANAs during ongoing regeneration was performed, splitting the ANAs into proximal and mid-distal graft segments. Transcriptome analysis of nerve gaps repaired using either short (2 cm) or long (4 cm) ANAs revealed modest differences in the expression profile of proximal grafts by 4 weeks post-surgery. Of the total genes expressed in short and long ANAs (> 15,000 genes), 127 genes were differentially expressed between these groups (Log_2_FC > 2 or < –2; adj. *P* value < 0.05). Of these differentially expressed genes (*DEG*), 33 genes were upregulated, and 94 genes downregulated in the proximal graft region of long ANAs relative to short ANAs (**Figure 1A**). Several of the upregulated genes have been associated with phagocytosis and antigen recognition (*AABR07034739.3*, *AABR07060872.1*, *AABR07065768.1*, *AABR07065823.2*, and *Ighm*). Conversely, within the downregulated genes, several are associated with axonal transport (*Mal*, *Nfasc*, and *Ugt8*) and positive regulation of ion transporter activity (*Adipoq*, *Gal*, *Kcna1*, *Kcnj11*, *Kcnk3*, and *Lrrc55*). Measurement of gene expression levels revealed that among the upregulated DEG, *AABR07060872.1*, *Ighm*, and *Krt75* were the highest expressed genes. For downregulated DEG, *Mal* and *Mpz* were highly expressed in the samples (**Figure 1B**).

To understand the changes in cellular and molecular pathways, Gene Ontology (GO) Biological Process, KEGG pathways, and molecular function analyses were performed. Based on the expression profile of all genes, GO Biological Process analysis revealed upregulation of immune response and catabolic processes in the long ANAs relative to short ANAs. Conversely, processes associated with nervous system development and cell-cell signaling were downregulated in the long ANAs (**Figure 1C**). Interestingly, KEGG pathways analysis revealed upregulation of pathways associated with lysosomal activity, autophagy, oxidative phosphorylation and immune cell differentiation and downregulation of pathways associated with protein digestion, absorption, interactions between cells and ECM, neuroactive ligand activation and the PI3K-Akt signaling pathway in the long ANA relative to short ANA (**Figure 1D**). Lastly, molecular function analysis showed downregulation of transmembrane channel activity and ECM structural molecules in the long ANAs (**Figure 1E**). These results demonstrate that the microenvironment in the proximal graft region of long ANAs is immunologically and metabolically more active and less regenerative compared with short ANAs by 4 weeks post-surgery.

### The transcriptome differs in the mid-distal graft region of acellular nerve allografts based on acellular nerve allograft length

**Figure 2:**
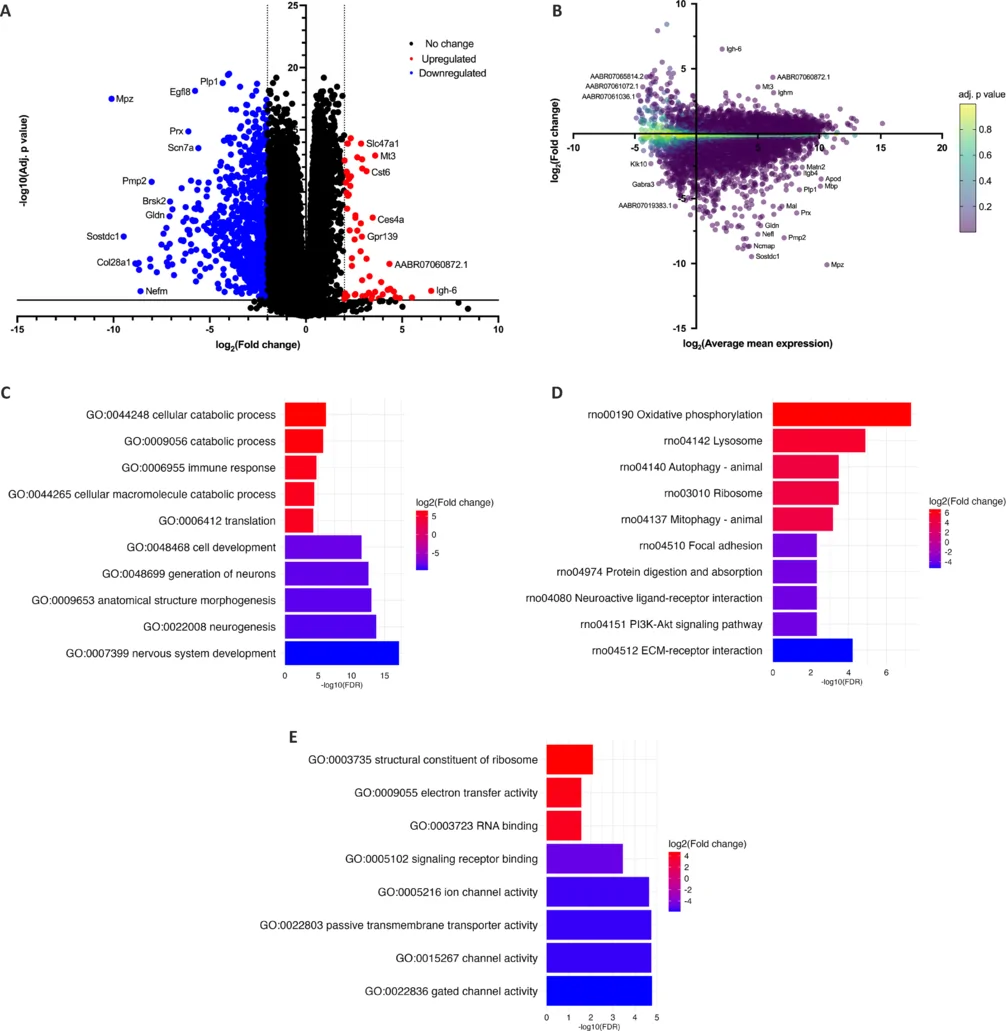
The mid-distal graft of long *versus* short ANAs is characterized by upregulation of autophagy, metabolic, catabolic, and immunological processes, and downregulation of processes associated with nerve regeneration. (A) Volcano plot representing genes expressed in the long *versus* short ANAs. Black dots represent non-DEGs, blue dots represent down-regulated DEGs and red dots upregulated DEGs in long ANAs *versus* short ANAs. (B) Log ratio *versus* average (MA) plot representing long ANAs *versus* short ANAs. Classification of top five upregulated and downregulated (C) GO biological processes, (D) KEGG pathways, and (E) Molecular function for long *versus* short ANAs. *n* = 8 per group, where *n* is each data point representing an animal. ANAs: Acellular nerve allografts; DEGs: differentially expressed genes; GO: gene ontology; KEGG: Kyoto Encyclopedia of Genes and Genomes.

Transcriptome analysis of the same short *versus* long ANAs from the previous section, now considering the mid-distal graft of ANAs, revealed 819 DEG between groups. 59 DEG were upregulated and 760 downregulated in the long ANAs relative to the short ANAs (**Figure 2A**). Similar to the proximal graft, several of the upregulated DEG have been associated with phagocytosis, antigen recognition and complement activation (*AABR07065768.1*, *AABR07065823.2*, *AABR07065837.1*, *AABR07065883.1*, *Ighm*, and *Cfp*) while several downregulated DEG have been associated with peripheral nervous system development, glial cell differentiation and myelination (*Errb2*, *Errb3*, *Gdnf*, *Mal*, *Mpz*, *Ngf*, *Plp1*, *Prx*, *Sox6*, *Sox8*, and *Sox10*). Measurement of gene expression levels revealed that among upregulated DEG *AABR07060872.1*, *Igh-6*, *Ighm*, and *Mt3* were the highest expressed genes in the groups. For downregulated DEG *Apod*, *Egfl8*, *Gldn*, *Itgb4*, *Mal*, *Matn2*, *Mbp*, *Mpz*, *Ncmap*, *Nefl*, *Plp1*, *Pmp2*, and *Sostdc1* were amongst the highest expressed genes in the groups (**Figure 2B**).

To further understand the microenvironment of ANAs, GO Biological Process analysis revealed upregulation of catabolic processes, immune activation and translation, and downregulation of nervous system development, neurogenesis, and morphogenesis in the mid-distal graft of long *versus* short ANAs (**Figure 2C**). Similarly, KEGG pathways analysis revealed upregulation of oxidative phosphorylation, lysosomal pathways, autophagy, mitophagy, and ribosomal assembly in the long ANAs. Conversely, PI3K-Akt signaling pathway, ECM-cell receptor interaction, neuroactive ligand receptor interaction, and protein digestion and absorption, were downregulated in the long ANAs relative to the short ANAs (**Figure 2D**). Molecular function analysis revealed upregulation of RNA binding, ribosomal structural constituents, and electron transfer activity and downregulation of transmembrane transport and ion channel activity in the mid-distal graft of long versus short ANAs (**Figure 2E**). Taken together, these results suggest the mid-distal microenvironment of long ANAs is metabolically more degradative, immunologically more active and less regenerative compared with the short ANAs.

### FK506 improves axonal regeneration and changes blood vessels

**Figure 3:**
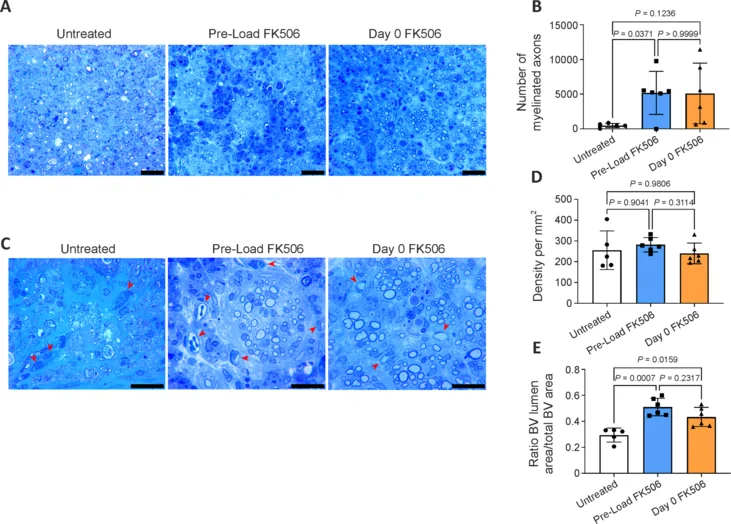
Histomorphometric analyses of the mid-distal graft region of long ANAs at 8 weeks post-surgery. (A, B) Representative histological images of graft cross-sections containing myelinated axons (A) and quantification of the number of myelinated axons (B). (C) Representative histological images of graft cross-sections showing BVs (red arrowheads). (D, E) Quantification of BV density (D) and BV morphology (E). Scale bars: 15 µm. Data represented as mean ± SD with each data point representing an animal (*n* = 5 in the untreated group; *n* = 6 in the pre-load tacrolimus (FK506) group; *n* = 6 in the day 0 FK506 group). *P* values are represented above each comparison, using one-way analysis of variance with the Tukey’s comparisons for *post hoc* analysis between groups. ANAs: Acellular nerve allografts; BV: blood vessel.

Based on previous work showing the development of a pro-inflammatory environment across the mid-distal graft of long ANAs (Acevedo Cintron et al., 2024), and the current transcriptional analysis revealing upregulation of immune pathways in these longer grafts, we used tacrolimus (FK506) to modulate the immune response and potentially regeneration across long ANAs. In this assessment, we used histomorphometric analysis to assess the extent of nerve regeneration within the mid-distal region of long ANAs by 8 weeks post-surgery (**Figure 3** and **Additional Figure 4**). Both, a preload and day of surgery FK506 treatment were used to observe the effects different dosing regimens have on nerve regeneration across long ANAs. Assessment of myelinated axon counts in the mid-distal graft of long ANAs treated with FK506 3 days before injury (pre-load FK506) revealed a 10-fold increase in the number of myelinated axons in comparison to the untreated group (**Figure 3B**). Similarly, pre-load FK506 caused a 5.7-fold increase in the area of the graft populated by myelinated axons and reduced the amount of axonal debris by 32% compared with the untreated group (**Additional Figure 4C** and **E**). Compared with the untreated group, the FK506 group treated on the day of injury (day 0 FK506) trended towards a 10-fold increase in the number of myelinated axons in the mid-distal graft region (**Figure 3B**). When comparing both FK506 treatment groups, we did not observe differences in regeneration. These results suggest FK506 can improve regeneration in the mid-distal graft region of long ANAs.

To further assess changes in the regenerative microenvironment of long ANAs, we used histomorphometric analysis to identify the effects of FK506 on the density and morphology of blood vessels within long ANAs at 8 weeks post-surgery. Previously, we reported no differences in the density of blood vessels, but a 25% decrease in the lumen to blood vessel area within the mid-distal graft region of long ANAs in comparison to short ANAs (Acevedo Cintron et al., 2024). In our current study, we observed that treatment with both pre-load and day 0 FK506 did not affect the density of blood vessels (**Figure 3D**). However, assessment of the morphology of blood vessels within the mid-distal grafts showed treatment with pre-load FK506 prevents by 21% the decrease in the lumen to blood vessel area observed in the blood vessels within the mid-distal graft of untreated ANAs (**Figure 3E**). Similarly, the Day 0 FK506 treatment prevents by 14% the decrease in the lumen to blood vessel area observed in the untreated ANAs (**Figure 3E**). The lumen to blood vessel area did not differ between the pre-load FK506 and day 0 FK506. Although the decrease in the number of myelinated axons coincides with changes in blood vessel morphology, correlation analysis revealed no monotonic relationship between these two variables (**Additional Figure 4K**). Taken together, these results demonstrate FK506 dosing strategy prevents changes in blood vessel morphology within ANAs.

### FK506 promotes motor functional recovery

**Figure 4:**
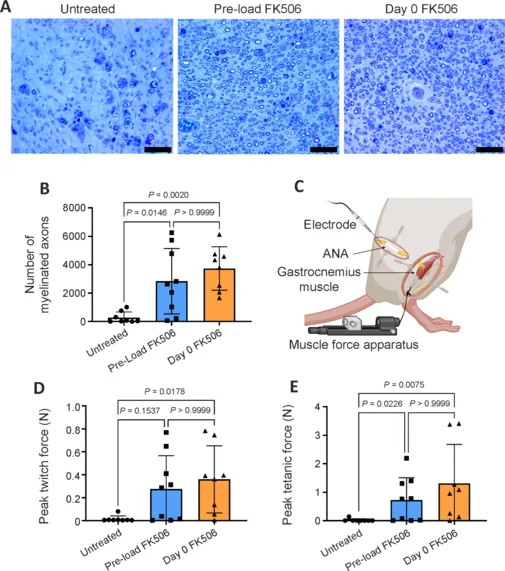
Tacrolimus (FK506) increases the number of myelinated axons regenerating across long ANAs and improves motor functional recovery at 12 weeks post-surgery. (A, B) Representative histological images of distal nerve cross-sections (A) and quantification of the number of myelinated axons (B). (C–E) Representation of muscle force apparatus (C), with quantification of peak twitch force (D) and peak tetanic force (E) of gastrocnemius muscle. Scale bars: 15 µm. Data represented as mean ± SD with each data point representing an animal (*n* = 8 in the untreated group; *n* = 9 in the pre-load FK506 group; *n* = 8 in the day 0 FK506 group). *P* values are represented above each comparison, using one-way analysis of variance with the Tukey’s comparisons for *post hoc* analysis between groups. Figure 4C was created with BioRender.com. ANAs: Acellular nerve allografts.

As a hallmark of successful regeneration, we assessed the number of myelinated axons in the distal nerve of FK506 treated and untreated long ANAs by 12 weeks post-surgery (**Figure 4** and **Additional Figure 5**). We observed myelinated axons to increase by 10-fold for the pre-load FK506 treated and 13.2-fold for the Day 0 FK506 treated compared with untreated (**Figure 4B**). Beyond axon counts, additional histomorphometric analyses revealed improved nerve regeneration with FK506. The pre-load FK506 group compared with the untreated group increased the percent of nerve tissue by 10-fold, percent of myelinated axons by 10-fold, area of the graft occupied by myelinated axons by 11.6-fold, and the density of myelinated axons by 8.9-fold (**Additional Figure 5A–D**). In addition, the pre-load FK506 groups contained a higher proportion of larger axons (6–7 μm) (**Additional Figure 5K**). Similarly, the Day 0 FK506 group increases the percent of nerve tissue by 13-fold, percent of myelinated axons by 13-fold, area of the graft covered by myelinated axons by 10.4-fold, density of myelinated axons by 8.9-fold and area of myelin covering the axons by 1.30-fold (**Additional Figure 5A–D**, **J**). The day 0 FK506 treatment also increased the proportion of larger axons (4–7 μm) (**Additional Figure 5K**). The comparison between the pre-load FK506 *versus* the day 0 FK506 showed no differences in nerve regeneration.

Assessment of twitch and tetanic force generated by the gastrocnemius muscle measured 12 weeks post-surgery revealed improved motor recovery with FK506. Measurements revealed that day 0 FK506 increased peak twitch force by 24.3-fold compared with untreated (**Figure 4D**). The pre-load FK506 trended towards an 18.6-fold increase in peak twitch force in comparison to untreated. Similarly, both the pre-load FK506 and day 0 FK506 groups increased peak tetanic force by 31.2-fold and 56.3-fold, respectively, relative to untreated (**Figure 4E**). Additionally, accounting for potential muscle mass differences, day 0 FK506 still revealed increased peak twitch force per gastrocnemius muscle weight by 16.4-fold, while both the pre-load FK506 and day 0 FK506 groups still had increased peak tetanic force per gastrocnemius muscle weight by 27-fold and 40.2-fold, respectively, compared with the untreated (**Additional Figure 5L** and **N**). Assessment of the relative mass weight of the gastrocnemius muscle revealed that the pre-load FK506 trended towards a 1.5-fold increase in relative mass weight in comparison to untreated. For the Day 0 FK506, there was a 1.6-fold increase in the relative mass weight of the gastrocnemius muscle in comparison to untreated (**Additional Figure 5P**). There were no differences in gastrocnemius muscle force or relative mass weight between the pre-load FK506 and Day 0 FK506. Finally, we assessed the correlation between the number of myelinated axons, and twitch and tetanic force, respectively. This analysis revealed a strong positive correlation between the number of myelinated axons and twitch force in the pre-load group (**Additional Figure 5M**). For tetanic force, analysis revealed a strong positive correlation between the number of myelinated axons and tetanic force in the untreated and pre-load groups (**Additional Figure 5O**).

To assess sensory recovery, we measured touch and pressure sensation using mechanical sensitivity analyses, but no differences were observed amongst groups (**Additional Figure 5Q**). These results revealed that FK506 can promote regeneration and reinnervation of motor targets across long ANAs.

### Treatments targeting individual pathways of FK506 signaling are not sufficient to improve regeneration

**Figure 5:**
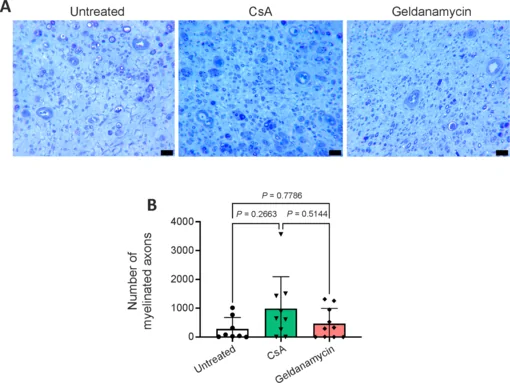
CsA or geldanamycin does not modify regeneration across long acellular nerve allografts. (A, B) Representative histological images of distal nerve cross-sections at 12 weeks post-surgery (A) with quantification of the number of myelinated axons (B). Scale bars: 15 µm. Data are expressed as mean ± SD with each data point representing an animal (*n* = 8 in the untreated group; *n* = 8 in the CsA group; *n* = 9 in the geldanamycin group). *P* values are represented above each comparison, using one-way analysis of variance with the Tukey’s comparisons for *post hoc* analysis between groups. CsA: Cyclosporine.

To further elucidate the mechanism by which FK506 promotes regeneration across long ANAs, we used two drugs that share similar known mechanisms to those of FK506. FK506 targets both an immunosuppressant (via calcineurin) and regenerative (steroid receptor complex) pathway (Lee et al., 2000; Barbarino et al., 2013; Konofaos and Terzis, 2013; Saffari et al., 2019). Cyclosporine is an immunosuppressant that targets the calcineurin pathway akin to FK506, while geldanamycin promotes regeneration via the steroid receptor complex pathway akin FK506 (Krönke et al., 1984; Bain et al., 1988; Midha et al., 1992, 2019; Mouzaki et al., 1992; Gold, 1999; Gold et al., 1999; Sun et al., 2012). We studied the effects of these drugs on nerve regeneration across long ANAs at 12 weeks through histomorphometric analyses, which revealed minimal improvements to axon regeneration compared with untreated. Histomorphometry analysis of myelinated axons counts within the distal nerve showed no difference between the cyclosporine-treated or geldanamycin-treated compared with untreated (**Figure 5**). These data suggest that targeting a single pathway mediated from FK506 administration alone is not sufficient to promote nerve regeneration across long ANAs.

### Tacrolimus (FK506) causes changes in the transcriptome of long acellular nerve allografts

Based on the previous results revealing FK506 improves nerve regeneration across long ANAs, we used the day 0 FK506 dosage treatment to identify changes in the transcriptome of long ANAs treated with FK506. The rationale for using the day 0 FK506 dosage regimen versus the pre-load FK506 was because the day 0 FK506 group showed superior regeneration compared with the untreated group yet caused fewer side effects (**Figure 4** and **Additional Figure 2**). Transcriptome analysis 4 weeks post-surgery at the proximal graft region revealed FK506 had minimal changes to gene expression. Of the total genes expressed (> 13,000), only 13 were DEG. Of these, 5 DEG were upregulated and 8 DEG downregulated in the FK506 relative to the untreated (**Additional Figure 6A**). None of these genes were highly expressed (**Additional Figure 6B**). GO Biological Process, KEGG pathways, and molecular function analyses revealed upregulation of oxygen transport and assembly of ribosomes processes in the proximal graft region of FK506 versus untreated (**Additional Figure 6C–E**).

**Figure 6:**
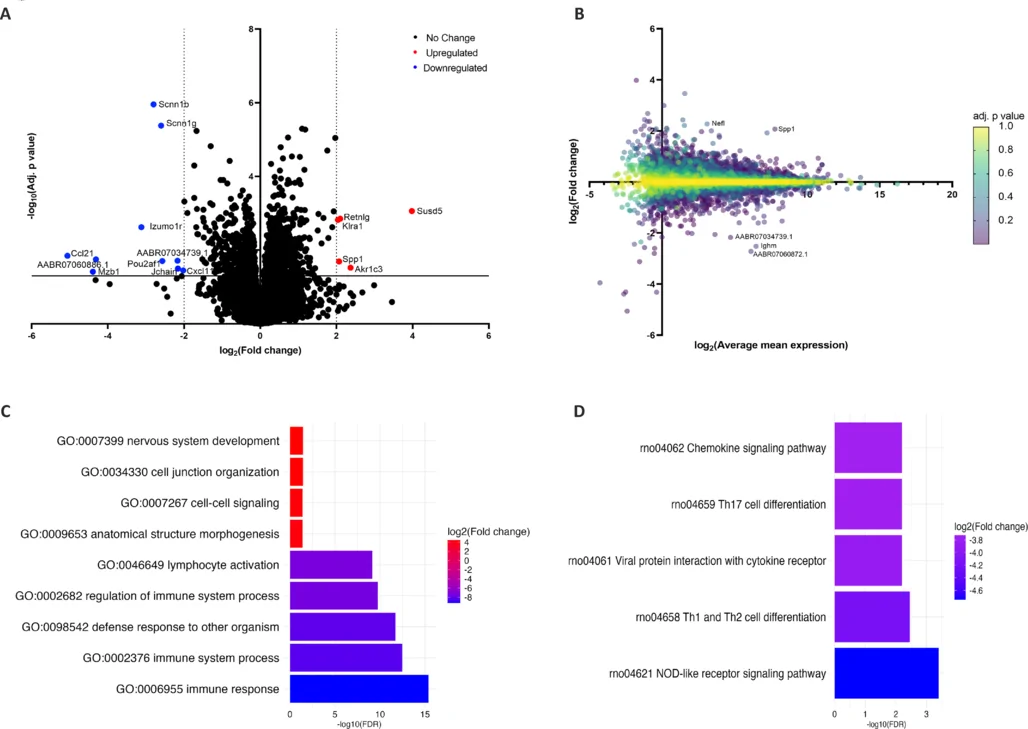
Tacrolimus (FK506) downregulates immune activation processes and upregulates processes associated with nerve regeneration in the mid-distal graft region of long ANAs. (A) Volcano plot representing genes expressed in the FK506 treated *versus* untreated long ANAs. Black dots represent non-DEGs, blue dots represent down-regulated DEGs and red dots upregulated DEGs in the FK506 treated *versus* untreated ANAs. (B) Log ratio *versus* average (MA) plot representing FK506 treated *versus* untreated groups. (C, D) Classification of top five upregulated and downregulated (C) GO biological processes and KEGG pathways (D) for FK506 treated and untreated long ANAs. *n* = 7 in the untreated group; *n* = 8 in the FK506 group, where *n* is each data point representing an animal. ANAs: Acellular nerve allografts; DEGs: differentially expressed genes; GO: gene ontology; KEGG: Kyoto Encyclopedia of Genes and Genomes.

Transcriptome analysis of the mid-distal graft region of FK506 *versus* untreated long ANAs revealed only 15 DEG between groups. Of these, 5 DEG were upregulated and 10 DEG downregulated for FK506 relative to untreated (**Figure 6A**). Amongst the upregulated genes, *Spp1* was the only gene that was highly expressed. For the downregulated genes, *AABR07034739.1* was highly expressed in the groups (**Figure 6B**). GO Biological Process analysis revealed FK506 upregulates processes associated with nervous system development, cell junction organization, and cell–cell signaling, while downregulates processes associated with the immune response, immune cell regulation, and lymphocyte activation (**Figure 6C**). Similarly, KEGG pathways analysis revealed FK506 downregulates pathways associated with T cell differentiation, NOD-signaling, and chemokine signaling pathways (**Figure 6D**). Taken together, these results revealed that FK506 alters the microenvironment of the mid-distal graft region in long ANAs by upregulating regenerative processes and downregulating processes related to immune cell activation.

### FK506 increases Schwann cells and macrophages within long acellular nerve allografts

**Figure 7:**
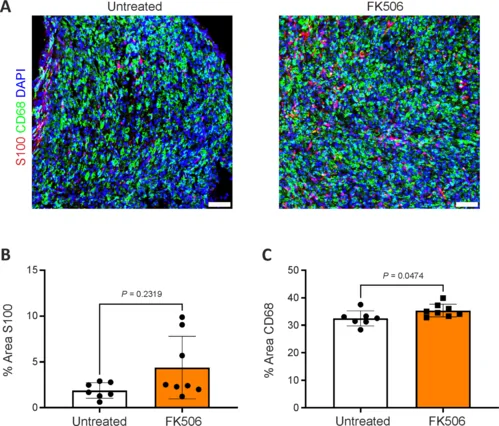
Tacrolimus (FK506) increases expression of macrophage markers at the mid-distal graft region of long acellular nerve allografts at 4 weeks post-surgery. (A) Immunofluorescence images of graft cross-sections showing Schwann cells (S100; red) and macrophages (CD68; green). (B, C) Quantification of graft cross-sections for percent area stained S100 (B) and CD68 positive (C). White scale bar is 30 µm (20× magnification). Data represented as mean ± SD with each data point representing an animal (*n* = 7 in the untreated group; *n* = 8 in the FK506 group). *P* values are represented above each comparison, using unpaired *t*-tests with Welch’s correction. DAPI: 4′,6-Diamidino-2-phenylindole.

To further understand the effect of FK506 on regeneration, we considered cell populations present within long ANAs. Interestingly, there was a 50% increase in S100^+^ (SC) area within the proximal graft of FK506 *versus* untreated, but no difference in CD68^+^ (Macrophage) area (**Additional Figure 7**). Within the mid-distal graft region, we observed a 10% increase in CD68^+^ area but no difference in S100^+^ area in the FK506 *versus* untreated (**Figure 7**). However, we identified a trend in the mid-distal graft region of FK506 where we observed a 2.3-fold increase in S100^+^ area relative to untreated (**Figure 7B**). These results demonstrate that FK506 affects the cellular composition across long ANAs.

### FK506 increases mRNA expression, but not protein expression, of *Spp1* in Schwann cells

**Figure 8:**
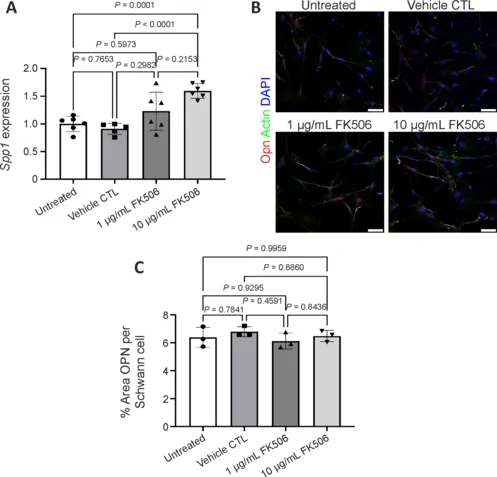
Tacrolimus (FK506) increases *Spp1* gene expression, but not Osteopontin protein expression from cultured SCs. (A) Quantification of mRNA levels of *Spp1* from SCs *in vitro*. (B) Immunofluorescence images of FK506 treated and untreated SCs. (C) Quantification of percent area stained Spp1 positive per cell. White scale bar is 50 µm (60× magnification). Data represented as mean ± SD (*n* = 6/group for quantitative reverse transcription-polymerase chain reaction except for Vehicle CTL with *n* = 5; *n* = 3/group for immunofluorescence). *P* values are represented above each comparison, using one-way analysis of variance with the Tukey’s comparisons for *post hoc* analysis between groups. All experiments were performed in three independent biological replicates. CTL: Control; DAPI: 4’,6-diamidino-2-phenylindole; OPN: osteopontin; SCs: Schwann cells.

Based on the transcriptome analysis of the mid-distal graft regions treated with FK506, we identified *Spp1* as a potential candidate involved in improved regeneration across long ANAs (**Figures 2B** and **6B**). Previous studies have shown *Spp1* is expressed in macrophages and SCs, therefore, we treated cultured SCs *in vitro* with different concentrations of FK506 for 48 hours to determine the effect on *Spp1* expression (Rentsendorj et al., 2018; Wang et al., 2020; Cappellano et al., 2021; Kaleta, 2021). After treatment, qRT-PCR data showed FK506 at a concentration of 10 µg/mL increases mRNA expression of *Spp1* by 60% (**Figure 8A** and **B**). However, protein quantification using immunohistochemistry did not show differences between groups (**Figure 8C**). These results suggest that FK506 can affect the mRNA expression levels of *Spp1*.

## Discussion

To improve regeneration across long ANAs (> 3 cm), we used transcriptome analysis to better understand which factors promote or inhibit regeneration across ANAs, as well as consider if FK506 administration impacted nerve regeneration. Through these studies, we identified differences in the regenerative environment of short and long ANAs suggesting modulation of the immune system could benefit regeneration across long ANAs. Furthermore, the immunosuppressive drug FK506 can indeed promote and rescue regeneration across even long (4 cm) ANAs.

Previously, our group has shown the development of a pro-inflammatory environment in long (4 cm) ANAs in contrast to the regenerative environment in short (2 cm) ANAs 4 weeks post-surgery (Acevedo Cintron et al., 2024). Our present study aims to identify earlier differences in the microenvironment of short and long ANAs before the development of the pro-inflammatory environment. Transcriptome analysis of the long ANAs (proximal and mid-distal graft) 4 weeks post-surgery revealed upregulation of autophagic, catabolic, immune, and oxidative phosphorylation pathways in contrast to the microenvironment of short ANAs that was characterized by upregulation of regenerative pathways. Upregulation of these pathways suggests increased energy demand and tissue remodeling across the long ANAs at this timepoint. Interestingly, later in the regenerative process, regeneration across the proximal graft of long ANAs is robust suggesting a shift from an immune activated and catabolic state to a regenerative one. In contrast, regeneration across the mid-distal graft of long ANAs is poor or limited suggesting a failure to shift from a degenerative and immune activated state to a regenerative one (Acevedo Cintron et al., 2024). Interestingly, previous studies have shown the differences in the transcriptome profile between the mid-distal graft of short and long ANAs observed in this study are preceded by an upregulation of immune activation in the mid-distal graft of short versus long ANAs (Pan et al., 2019). Then, by 4 weeks post-surgery, there is a decrease in the number of macrophages and T cells in the mid-distal graft of long *versus* short ANAs (Pan et al., 2019). At this timepoint, the mid-distal graft of long ANAs becomes degradative and immunologically more active in comparison to the short ANAs. Given the presence of more macrophages and T cells in the short (which upregulates pro-regenerative process) versus long (which upregulates degradative and immune processes) ANAs, it is possible that the subpopulations of immune cells present in the mid-distal graft region of short ANAs differ in their phenotype and function from those present in the mid-distal graft of long ANAs. Although it is unknown which cells are responsible for these differences in the transcriptome profile and microenvironment of short versus long ANAs, we hypothesize dysregulation of immune cells, and their phenotype contributes to the upregulation of catabolic processes and the subsequent development of a pro-inflammatory environment. Overall, these results suggest early remodeling and inflammation in the short ANAs is followed by activation of regenerative pathways (starting 4 weeks post-surgery), while in the long ANAs, early remodeling and inflammation are followed by the development of a continuous pro-inflammatory state.

We used the immunosuppressant tacrolimus (FK506) to observe the effect immunosuppression has on nerve regeneration across long ANAs. Although FK506 is known as an immunosuppressant, previous studies have demonstrated its effect in promoting nerve regeneration after injury (Udina et al., 2002; Yang et al., 2003; Snyder et al., 2006; Konofaos and Terzis, 2013; Davis et al., 2019; Jo et al., 2019; Saffari et al., 2019; Tajdaran et al., 2019). In our studies on long ANAs, FK506 prevented changes in blood vessel morphology (mid-distal graft), promoted nerve regeneration (mid-distal graft and distal nerve), and improved motor functional recovery. Transcriptome analysis revealed that FK506 prevented signaling related to T cell activation and promoted signaling related to nervous system development and cell junction organization. Interestingly, it has been shown that depletion of T cells across short ANAs disrupts and delays nerve regeneration (Pan et al., 2019). It is possible that the pro-regenerative effects of FK506 compensate for the lack of T cell activation or the effects of FK506 on T cell varies depending on the T cell subtype and activation state (Tocci et al., 1989). Additionally, this could mean that T cells have context-dependent roles, as short ANAs develop an anti-inflammatory environment that could be amplified via T cells, while long ANAs fail to transition to an anti-inflammatory environment that could be exacerbated by T cells. In addition to changing the transcriptome of long ANAs, FK506 affects the cellular composition of long ANAs by increasing the expression of SC (proximal graft) and macrophage (mid-distal graft) markers across these ANAs. Interestingly, a recent study demonstrated that FK506 increases the number of macrophages at the neuromuscular junction after nerve injury. Moreover, macrophages at the neuromuscular junction express FKBP-52 and disruption of macrophage infiltration diminishes the neuroenhancing effects of FK506 suggesting a role of macrophages in mediating the pro-regenerative effects of FK506 (Jablonka-Shariff et al., 2025). Overall, our results suggest some of the mechanisms by which FK506 improves regeneration across long ANAs are by increasing the number of macrophages and SCs while preventing T cell activation.

To elucidate which FK506-related mechanism, immunosuppression or promoting axonal growth, contributes more to regeneration across long ANAs, we used the drugs cyclosporine and geldanamycin, respectively. Cyclosporine is an immunosuppressant that prevents T cell activation by inhibiting calcineurin activation, while geldanamycin promotes axonal growth by upregulating GAP-43 and c-JUN within axons via a steroid receptor complex pathway (Bain et al., 1988; Mouzaki et al., 1992; Gold et al., 1999; Gold and Villafranca, 2003; Yu et al., 2009; Barbarino et al., 2013; Liddicoat and Lavelle, 2019; Migita et al., 2019). In this study, neither drug improved nerve regeneration across long ANAs comparable to FK506, suggesting that possible synergy between both pathways is necessary to promote nerve regeneration across long ANAs. In addition, it is possible FK506 promotes neurite elongation through other pathways besides the FKBP-52/steroid receptor complex pathway which is the only known pathway by which geldanamycin promotes neurite elongation. FK506 immunosuppressive and regenerative properties could also be more potent compared with cyclosporine and geldanamycin, respectively, since previous studies have suggested FK506 to be a stronger immunosuppressant in comparison to cyclosporine (Tocci et al., 1989; Sigal et al., 1991; Peterson et al., 1998).

Although FK506 improves nerve regeneration, its use in the clinic is limited due to undesirable side effects (Barbarino et al., 2013; Saffari et al., 2019). Therefore, we aimed to identify potential targets by which FK506 acts to promote nerve regeneration. Based on the transcriptome analyses of the mid-distal graft region (where we have shown regeneration starts to dwindle in untreated long ANAs), we identified the gene, *Spp1*, expressed as the protein osteopontin, as a potential candidate. Osteopontin is a protein expressed by macrophages and SCs that has been associated with pro- and anti-inflammatory states (Jander et al., 2002; Cappellano et al., 2021; Kaleta, 2021). A previous study with *in vitro* macrophages has shown that increased osteopontin expression promotes an anti-inflammatory phenotype in these macrophages (Rentsendorj et al., 2018). In uninjured nerves, osteopontin is constitutively expressed by SCs (Jander et al., 2002; Ahn et al., 2004; Wang et al., 2020). After nerve injury, osteopontin expression increases for the first 2 weeks and then returns to pre-injury levels (Jander et al., 2002; Ahn et al., 2004; Wang et al., 2020). Interestingly, this increase in osteopontin expression is caused by SCs and not infiltrating macrophages (Jander et al., 2002). To further understand the role of osteopontin in nerve regeneration, experiments in osteopontin null mice revealed lack of osteopontin disrupts regeneration and decreases reinnervation to neuromuscular junctions, suggesting osteopontin is necessary in advancing the regenerative process of motor neurons (Wright et al., 2014). Our *in vitro* experiments on primary SCs showed FK506 increases mRNA expression of *Spp1* but not osteopontin expression. It is possible that longer incubation times with FK506 are needed to reveal changes at the protein expression level or changes in the protein expression of osteopontin in SCs are dependent on microenvironmental cues presented to these cells. Techniques such as time course protein expression or ribosomal affinity purification could further identify changes in protein expression of osteopontin. Recently, Smail et al. (2024) suggested addition of osteopontin into 1 cm ANAs polarizes macrophages into an anti-inflammatory phenotype, reduces expression of pro-inflammatory cytokines, and improves functional outcomes. However, whether osteopontin expression is sufficient to improve nerve regeneration across long ANAs and the mechanism remains to be elucidated.

A limitation of this study was observing how the transcriptome of different cell populations changes with FK506. Although we showed that FK506 changes the transcriptome of ANAs, it is unknown how FK506 affects specific cell populations present in the long ANAs and the interactions between these cell populations. Future work will aim to use single-cell RNA sequencing and spatial transcriptomics to identify changes in the transcriptome of specific cell populations present in long ANAs and explore the interactions between these cell populations. Moreover, these additional experiments will help elucidate how FK506 changes the transcriptional profile of specific cell populations within long ANAs, and which cells are expressing *Spp1* *in vivo*. This future work, however, should also consider one of the possible mechanisms by which FK506 improves regeneration across ANAs *in vivo*, which is by directly affecting the axons since previous studies have demonstrated that FK506 promotes neurite elongation *in vitro* (Gold and Villafranca, 2003; Gold and Zhong, 2004; Saffari et al., 2019, 2021). Another limitation of this study is the variability in the animal response to FK506 since not all the treated animals showed similar outcomes. This variability in regeneration, however, is intrinsic to the long ANA model, as similar variations are observed in human studies of regeneration across long ANAs (Carlson et al., 2018; Nietosvaara et al., 2019; Leckenby et al., 2020; Safa et al., 2020; Peters et al., 2023). Although in this study, we focused on early reinnervation of the muscle and sensory target organs, follow up experiments could assess the longer-term outcomes from FK506 administration using gait analysis, walking track assessment, and nerve conduction.

Overall, our study showed upregulation of degenerative pathways across long ANAs, which was accompanied by limited regeneration. Treatment with FK506 improved regeneration across long ANAs, prevented the changes in blood vessel morphology observed in poorly regenerated ANAs and altered the microenvironment of ANAs by increasing the number of macrophages and SC markers. A major takeaway from this study is that FK506 promotes regeneration by activating the immunosuppressive and pro-regenerative pathways, as immunosuppression (via calcineurin pathway) or neurite elongation (via steroid receptor complex pathway) alone was not enough to promote regeneration. Finally, our study identified Osteopontin as a possible candidate to further understand the regenerative process across long ANAs. Future work will focus on exploring the synergy between anti-inflammatory and pro-regenerative pathways and on modulating the expression of Osteopontin to study its effect and role in nerve regeneration.

## Additional files:

***Additional Figure 1:*** *Tacrolimus (FK506) can act as an immunosuppressant (A) and promote neurite growth (B).*

Additional Figure 1 Tacrolimus (FK506) can act as an immunosuppressant (A) and promote neurite growth (B).
FK506 immunosuppresses by binding FKBP12, preventing calcineurin activation. Calcineurin inactivation prevents NFAT dephosphorylation and subsequently T cell activation. To promote neurite growth, it is hypothesized FK506 binds to FKBP52 which promotes activation of Erk signaling pathway and expression of neurotrophic factors leading to neurite growth.

***Additional Figure 2:*** *Representation of rat sciatic nerve injury model where the nerve gap is repaired using ANAs.*

Additional Figure 2 Representation of rat sciatic nerve injury model where the nerve gap is repaired using acellular nerve allografts (ANAs). (A, B) The right sciatic nerve of Lewis rats is transected (A) and the gap is immediately repaired using ANAs (B).

***Additional Figure 3:*** *Changes in the weights of rats in the untreated, pre-load tacrolimus (FK506) and day 0 FK506 groups as time progresses.*

Additional Figure 3 Changes in the weights of rats in the untreated, pre-load Tacrolimus (FK506) and Day 0 FK506 groups as time progresses.
Blue (for pre-load FK506 group) and orange (for Day 0 FK506 group) arrows represent the start of FK506 administration, respectively. Black arrow represents the end of FK506 dosage regimen. *n* = 8 in untreated group; *n* = 9 in Pre-Load FK506 group; *n* = 8 in Day 0 FK506 group, where N represents data from an animal. Statistical analysis comparisons are represented above each time point using one-way analysis of variance with the Tukey’s comparisons for *post hoc* analysis between groups. ^a^*P* < 0.05, Untreated *vs*. Pre-load FK506; ^b^*P* < 0.05, Untreated *vs*. Day 0 FK506; ^c^*P* < 0.05, Pre-load FK506 *vs*. Day 0 FK506 comparison.

***Additional Figure 4:*** *Pre-load tacrolimus (FK506) increases the area of the graft occupied by myelinated axons and decreases axonal debris in the mid-distal graft of long ANAs 8 weeks post-surgery.*

Additional Figure 4 Pre-Load Tacrolimus (FK506) increases the area of the graft occupied by myelinated axons and decreases axonal debris in the mid-distal graft of long acellular nerve allografts (ANAs) 8 weeks post-surgery.
Histomorphometric quantification of ANA cross-sections for: (A) Percent area of graft with neural tissue; (B) Percent area of graft with myelinated axons; (C) Area of graft with myelinated axons; (D) Density of myelinated axons within graft; (E) Percent area of graft with axonal debris; (F) Cross sectional area of graft; (G) Axon/myelin ratio; (H) Area of myelinated axons; (I) Area of axons; (J) Area of myelin; and (K) Correlation matrix between blood vessel morphology and the number of myelinated axons. Data represented as mean ± SD with each data point representing an animal (*n* = 5 in untreated group; *n* = 6 in Pre-Load FK506 group; *n* = 6 in Day 0 FK506 group). R represents Spearman correlation coefficient, and it is above its corresponding *P* value. *P* values for comparison between groups are represented above each comparison using one-way analysis of variance with the Tukey’s comparisons for *post hoc* analysis between groups.

***Additional Figure 5:*** *Tacrolimus (FK506) improves nerve regeneration across long ANAs and promotes motor recovery 12 weeks post-surgery.*

Additional Figure 5 Tacrolimus (FK506) improves nerve regeneration across long acellular nerve allografts (ANAs) and promotes motor recovery 12 weeks post-surgery.
Histomorphometric quantification of distal nerve cross-sections for: (A) Percent area of graft with neural tissue; (B) Percent area of graft with myelinated axons; (C) Area of graft with myelinated axons; (D) Density of myelinated axons within graft; (E) Percent area of graft with axonal debris; F) Cross sectional area of graft; G) Axon/myelin ratio; (H) Area of myelinated axons; (I) Area of axons; (J) Area of myelin; and (K) Fiber size distribution. (L, N) Quantification of peak twitch force (L) and peak tetanic force by muscle weight (N). (M, O) Correlation matrix between number of myelinated axons versus twitch muscle force (M) or tetanic muscle force (O), respectively. (P) Muscle mass ratio of injured versus contralateral side. Data represented as mean ± SD with each data point representing an animal (*n* = 10 in untreated group; *n* = 12 in Pre-Load FK506 group; *n* = 9 Day 0 FK506 group). R represents Spearman correlation coefficient, and it is above its corresponding *P* value. *P* values for comparison between groups are represented above each comparison, using one-way analysis of variance with the Tukey’s comparisons for *post hoc* analysis between groups.

***Additional Figure 6:*** *Tacrolimus (FK506) promotes upregulation of pathways involved in translation in the proximal graft of long versus short ANAs.*

Additional Figure 6 Tacrolimus (FK506) promotes upregulation of pathways involved in translation in the proximal graft of long *versus* short ANAs.
(A) Volcano plot representing genes expressed in the FK506 treated versus untreated long ANAs. Black dots represent non differentially expressed genes (DEGs), blue dots represent down-regulated DEGs and red dots upregulated DEGs in the FK506 treated versus untreated ANAs. (B) Log ratio vs average (MA) plot representing FK506 treated versus untreated groups. (C-E) Classification of top 5 upregulated and downregulated gene ontology (GO) biological processes (C), Kyoto Encyclopedia of Genes and Genomes (KEGG) pathways (D), and Molecular function for FK506 treated and untreated long ANAs (E). *n* = 7 in untreated group; *n* = 8 in FK506 group, where n represents data from an animal. ANAs: Acellular nerve allografts.

***Additional Figure 7:*** *Tacrolimus (FK506) increases expression of SC marker at the proximal graft region of long ANAs at 4 weeks post-surgery.*

Additional Figure 7 Tacrolimus (FK506) increases expression of SC marker at the proximal graft region of long ANAs at 4 weeks post-surgery.
(A) Immunofluorescence images of graft cross-sections showing SCs (S100; red) and macrophages (CD68; green). (B, C) Quantification of graft cross-sections for percent area stained S100 (B) and CD68 positive (C). Scale bars: 30 μm (20× magnification). Data represented as mean ± SD with each data point representing an animal (*n* = 7 in untreated group; *n* = 8 in FK506 group). *P* values are represented above each comparison using unpaired *t*-tests with Welch’s correction. ANAs: Acellular nerve allografts; SC: Schwann cell.

***Additional Table 1:*** *Number of animals assigned per experiment.*

***Additional Table 2:*** *Antibodies used for immunohistochemistry.*

***Additional Table 3:*** *Quantitative reverse transcription-polymerase chain reaction primers used in this study.*

## Data Availability

*All relevant data are within the paper and its Additional* *files.*
